# Efficacy of rational emotive digital storytelling intervention on knowledge and risk perception of HIV/AIDS among schoolchildren in Nigeria: Erratum

**DOI:** 10.1097/MD.0000000000013848

**Published:** 2018-12-21

The authors have provided a correction in the article, “Efficacy of rational emotive digital storytelling intervention on knowledge and risk perception of HIV/AIDS among schoolchildren in Nigeria ”,^[1]^ which appeared in Volume 97, Issue 47 of *Medicine*.

The 2.5 Procedure should read: This study adopted a group randomized controlled trial design,^[24]^ which involved a pretest, posttest, and follow-up evaluation. The intervention type consisted of an experimental group (REDStory) and a control group (wait-list control group). The study participants were randomly assigned to either wait-list control group or experimental group by 3 of the researchers.
